# The Translation of Cyclin B1 and B2 is Differentially Regulated during Mouse Oocyte Reentry into the Meiotic Cell Cycle

**DOI:** 10.1038/s41598-017-13688-3

**Published:** 2017-10-26

**Authors:** Seung Jin Han, João Pedro Sousa Martins, Ye Yang, Min Kook Kang, Enrico Maria Daldello, Marco Conti

**Affiliations:** 1 Department of Biological Sciences, Inje University, Gimhae, 50834 Republic of Korea; 2 Center for Reproductive Sciences, University of California, San Francisco, CA 94143 USA; 3 Eli and Edythe Broad Center of Regeneration Medicine and Stem Cell Research, University of California, San Francisco, CA 94143 USA; 4 Department of Obstetrics and Gynecology and Reproductive Sciences, University of California, San Francisco, CA 94143 USA; 5 State Key Laboratory of Agrobiotechnology, College of Biological Sciences, China Agricultural University, Beijing, 100193 People’s Republic of China

## Abstract

Control of protein turnover is critical for meiotic progression. Using RiboTag immunoprecipitation, RNA binding protein immunoprecipitation, and luciferase reporter assay, we investigated how rates of mRNA translation, protein synthesis and degradation contribute to the steady state level of Cyclin B1 and B2 in mouse oocytes. Ribosome loading onto *Ccnb1* and *Mos* mRNAs increases during cell cycle reentry, well after germinal vesicle breakdown (GVBD). This is followed by the translation of reporters containing 3′ untranslated region of *Mos* or *Ccnb1* and the accumulation of Mos and Cyclin B1 proteins. Conversely, ribosome loading onto *Ccnb2* mRNA and Cyclin B2 protein level undergo minimal changes during meiotic reentry. Degradation rates of Cyclin B1 or B2 protein at the GV stage are comparable. The translational activation of *Mos* and *Ccnb1*, but not *Ccnb2*, mRNAs is dependent on the RNA binding protein CPEB1. Inhibition of Cdk1 activity, but not Aurora A kinase activity, prevents the translation of *Mos* or *Ccnb1* reporters, suggesting that MPF is required for their translation in mouse oocytes. Conversely, *Ccnb2* translation is insensitive to Cdk1 inhibition. Thus, the poised state that allows rapid meiotic reentry in mouse GV oocytes may be determined by the differential translational control of two Cyclins.

## Introduction

Fully grown mammalian oocytes are arrested at the prophase of the first meiotic division through the activity of the cAMP-PKA dependent pathway^1,2^. Upon luteinizing hormone (LH) stimulation, signals from somatic cells relieve this cAMP-dependent cell cycle block and promote oocyte reentry into meiosis^3^. Loss of critical intercellular contacts and crosstalk with surrounding somatic cells induce oocyte meiotic reentry approximately 3 h after the hormonal signal *in vivo*, whereas removal of oocytes from the follicle causes meiotic reentry *in vitro* in 60–90 min. Meiotic resumption is dependent on the activity of the maturation promoting factor (MPF) whose properties have been elucidated mostly in the *Xenopus* oocyte model. MPF is a heterodimer composed of a Cyclin-dependent protein kinase 1 (Cdk1) and a Cyclin regulatory subunit, synthesized at different times during the cell cycle^4^. Networks of kinases and phosphatases, including c-Mos and Polo-like kinases, contribute to the fine tuning of the MPF activity through positive and negative feedbacks. In the mouse, two MPF regulators under PKA control, the inhibitory kinase Wee1B and the activating phosphatase Cdc25, participate in the regulation of the Cdk1/Cyclin complex^5,6^.

Given the virtual absence of transcription in fully grown oocytes, translation of maternal mRNAs accumulated earlier during development is the only form of gene expression that drives maturation in almost all species studied. This property of the female gamete has been explored in several model organisms including *Drosophila* and *Xenopus* ^7,8^. In frog oocytes, a large macromolecular complex including the RNA binding proteins (CPEB1, CPSF, ePABP), adenylase (Gld2), deadenylase (PARN), and the scaffold/adaptors Symplekin and Maskin, as well as components of the cap complex (eIF4E, 4E-T), prevents translation by maintaining the target transcripts with a short poly (A) tail^9,10^. Whether the same complex is functional in mammalian oocytes is unclear. When translation is activated in frog oocytes, CPEB protein becomes phosphorylated at Ser174 by Eg2/Aurora A kinase^11^, PARN is expelled from the repressive complex, the mRNA becomes polyadenylated, and ePAB binding to poly (A) promotes the loop conformation of the mRNA, ribosome recruitment, and translation^12,13^. This is thought to be responsible for a first wave of translation in frogs^11,12^. Some of the players and regulations are likely similar in mammalian and frog oocytes, but important differences are also present.

In frogs, translational activation of several key cell cycle regulators, including Mos and Cyclins, takes place when MPF activity is relatively low; this translation is necessary to prepare the *Xenopus* oocyte for meiotic reentry^14^. Conversely, the stockpile of Cyclins and Cdk1 proteins in mouse oocytes is sufficient for meiotic reentry without *de novo* protein synthesis^15^. However, the timing of this translational burst during mouse oocyte maturation has not been investigated and little is known about the molecular details of these translational controls in mammalian oocytes.

Synthesis and degradation of the Cyclins are finely tuned to maintain a stable, suspended dictyate state but at the same time allow rapid activation of Cdk1 when LH triggers cell cycle resumption. Accumulation of Cyclin B1 is prevented by degradation though ubiquitination via APC^Cdh1^ in GV oocytes^16^, whereas the proper amount of Cyclin B1 is maintained by Securin functioning as a competitor substrate for APC^Cdh1^ in GV oocytes^17^. Similarly, Cyclin B2 is degraded by APC^Cdh1^, however, the degradation is inhibited by the interaction with Hec1^18^. Although the amount of Cyclin B1 and Cyclin B2 is a key determinant for oocyte maturation, mechanisms regulating the translation of their mRNAs in quiescent mouse oocytes have not been explored thoroughly. We have used complementary experimental strategies, including a genome-wide approach, RNA immunoprecipitation (RIP) analysis, RiboTag immunoprecipitation assay, and reporter assay to examine the translation of key cell cycle components such as *Mos*, *Ccnb1* (gene for Cyclin B1 protein) and *Ccnb2* (gene for Cyclin B2 protein) during maturation of mouse oocytes. The different time courses and mechanisms of the Cyclins translation suggest temporally distinct functions for these important cell cycle regulators.

## Results

### Contrasting translation patterns of key cell cycle regulators are revealed by polysome array

**Figure 1:**
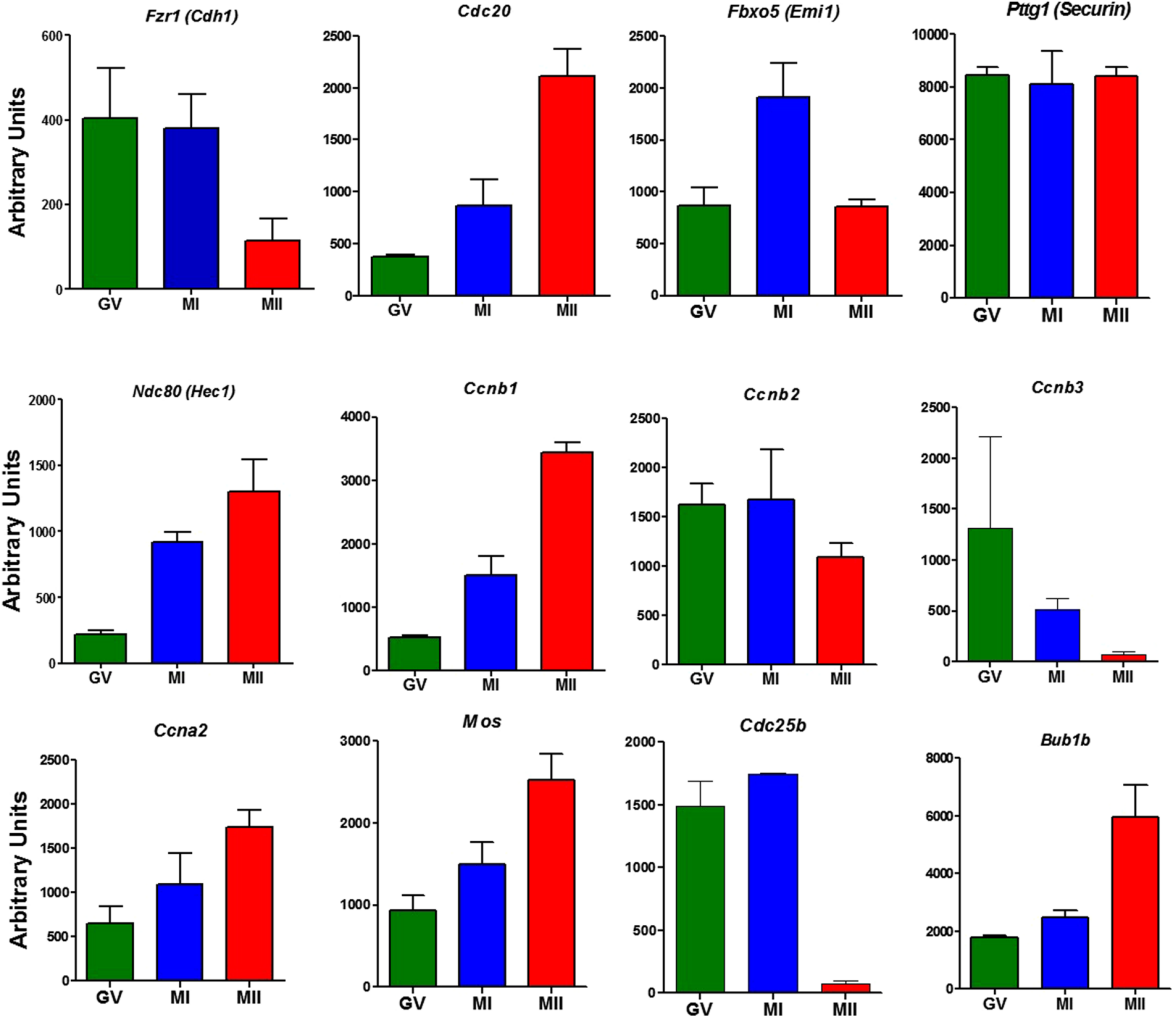
Differential polysome loading of transcripts coding for G2/M regulators. The association with ribosomes of transcripts coding for proteins known to be involved in the G2/M transition are reported for GV, MI, and MII. Each bar represents the mean ± SEM of three biological replicates. Note the divergent pattern of *Ccnb1* and *Ccnb2* mRNA association with the polysomes.

During *Xenopus* oocyte maturation, the temporal recruitment to the translation machinery of specific mRNAs is a pivotal regulatory mechanism required for meiosis reentry^19^. Conversely, the mechanism underlying the activation of translation during resumption of meiosis in mouse oocytes has not been investigated thoroughly. To define how translational regulations contribute to meiotic reentry in mouse oocytes, we mined the polysome-array data we have previously generated^20^. This data-set was generated by collecting mouse oocytes matured *in vivo* at the GV, prometaphase, and MII stages of development. Oocyte extracts were then fractionated by sucrose density gradients and polysomes used to extract mRNA. The mRNAs were then analyzed by microarray hybridization with the assumption that they would represent translating transcripts. Using this unbiased approach, we surveyed components known to be involved in the maintenance of GV as well as GV/GVBD transition. The translation of several mRNAs including *Cdc20*, *Ncd80 (Hec1)*, *Ccnb1*, *Mos*, *Bub1b*, and *Ccna2* increased progressively during maturation, while the translation of another set of transcripts (*Cdh1*, *Cdc25b*, *Ccnb3* and *Ccnb2*) declines or remains unchanged as the oocytes progress through meiosis (Fig. 1). Of note, the *Ccnb1* and *Ccnb2* mRNAs coding for Cyclin B1 and B2 proteins, which are the major components of MPF complex, clearly diverged in their translational pattern (Fig. 1), with *Ccnb1* mRNA being recruited to the polysomes at prometaphase whereas the *Ccnb2* mRNA association with the polysome increased only marginally or not at all during this transition. The association of *Ccnb3* mRNA with polysomes decreased progressively with maturation suggesting a decline in accumulation of this Cyclin (Fig. 1).

### Translation of *Ccnb1* and Mos, but not *Ccnb2*, transcripts is activated after GVBD

**Figure 2:**
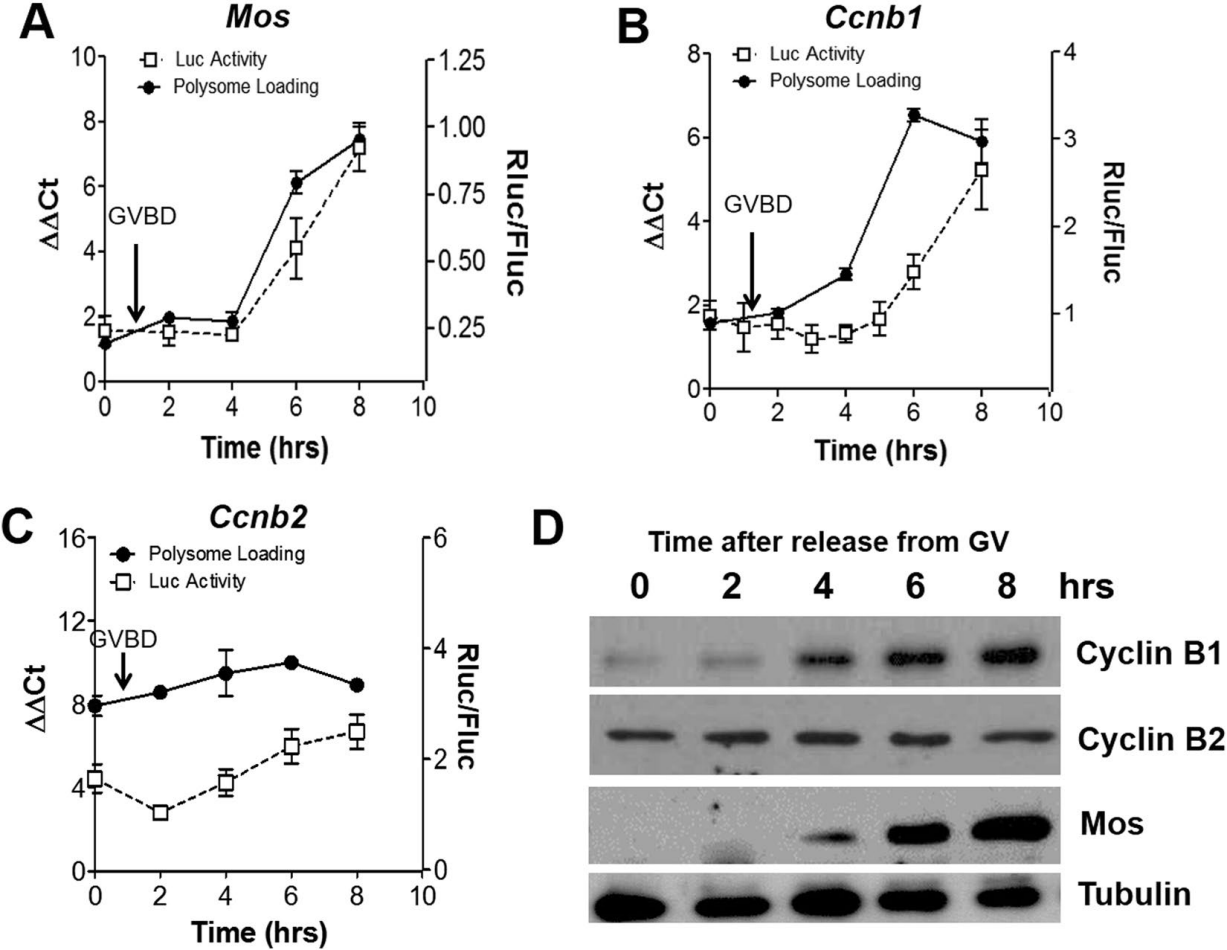
Ribosome loading onto *Mos*, *Ccnb1*, and *Ccnb2* mRNA and translation of reporters. Oocytes from RiboTag transgenic mice were isolated at the GV stage and matured *in vitro* for the indicated times. Extracts were then subjected to immunoprecipitation with HA antibody or IgG (RiboTag IP) and the recovery of transcripts in the IP pellet monitored by qPCR. The *Mos* (**A**), *Ccnb1* **(B**) and *Ccnb2* (**C**) mRNAs recovery in the IP pellet are reported as solid symbols. Each point is the mean ± SEM, N = 3. In parallel, oocytes from wild type mice were collected and injected with cRNAs coding for the Firefly luciferase and each of the reporters containing Renilla luciferase fused to the *Mos*, *Ccnb1* or *Ccnb2* 3′UTR. After recovery in media with milrinone, oocytes were released from cell cycle arrest by washing and transferring to media without milrinone. Oocytes were collected at the indicated time points and the accumulation of the luciferase reporter was measured (dashed lines). Each point is the mean ± SEM of at least 5 biological replicates. (**D**) Accumulation of the endogenous Mos, Cyclin B1 and Cyclin B2 proteins during oocyte maturation was monitored using western blot analysis. Extracts from 100 oocytes per lane were resolved on the SDS PAGE and proteins were detected with specific antibodies, respectively. Tubulin levels were monitored as the loading control. The experiment was repeated twice with identical results.

To increase the temporal resolution of these changes in translation, we investigated the ribosome loading onto these transcripts at multiple time points during prophase, GVBD, and prometaphase (Fig. 2). For this purpose, we used the RiboTag RIP strategy which has successfully been used to monitor ribosome loading of maternal mRNAs during oocyte maturation^21,22^. With this method, the HA-tagged ribosome protein (Rpl22) exclusively expressed in the oocyte in C57BL/6-Zp3cre-RiboTag mice was immunoprecipitated with HA-specific antibody from extracts of oocytes collected at several time points throughout meiotic progression. The mRNA recovered in the immunoprecipitation pellet were reverse transcribed and amplified, then either libraries for RNAseq were prepared or cDNA was used for qPCR. This approach requires 1/10 of the oocytes when compared to polysome array. Consistent with previously obtained polysome-array data^20^ (Fig. 1), we observed that ribosome loading on the *Ccnb2* transcript did not change significantly from the GV state to 8 h after resumption of meiosis in the RNAseq data (Suppl. Figure 1). Conversely, the amount of *Ccnb1* and *Mos* transcripts recovered in association with ribosomes increased significantly during maturation (Suppl. Figure 1). We then confirmed this divergent behavior of the *Ccnb1*, *Ccnb2* and *Mos* mRNAs by RiboTag/qPCR (Fig. 2). After immunoprecipitation of the HA-tagged ribosome complex, we performed quantitative PCR using primers specific for *Ccnb1*, *Ccnb2*, and *Mos* respectively. The ribosome loading on *Mos* and *Ccnb1* mRNAs increased from 2–4 h up to 6–8 h (Fig. 2A,B). Conversely, the ribosome loading onto the *Ccnb2* mRNA increased only marginally and in an insignificant way during the same time frame (Fig. 2C).

The 3′ untranslated region (3′UTR) of a gene is critically involved in the regulation of translation by RNA binding proteins through control of the length of poly(A) tail. To test whether the ribosome loading onto the transcripts recapitulates 3′UTR-dependent translation, we constructed reporters by fusing the Renilla luciferase coding region with 3′UTR of mouse *Mos*, *Ccnb2* or *Ccnb1* genes. We injected these reporter constructs together with the same amount of Firefly luciferase reporter, which is expressed at a constant level, and that was used a control. After collection of the oocytes at the indicated time points, the ratio between accumulated Renilla luciferase and Firefly luciferase was calculated. The increased accumulation of the reporter followed the ribosome loading onto the *Mos* and *Ccnb1* transcripts (Fig. 2A,B). Again, *Ccnb2* mRNA ribosome loading and reporter translation were marginally affected during maturation (Fig. 2C). Differences in initial rates of translation of the *Ccnb1* and *Ccnb2* reporters were confirmed by additional experiments where the reporter accumulation was measured from 30 min after mRNA injection in oocytes maintained in GV. The rate of *Ccnb2* reporter translation was significantly higher than that of *Ccnb1* (Suppl. Figure 2).

To confirm that the changes in translation rate, if present, corresponded to changes in endogenous protein accumulation, western blots were performed at different times during oocyte maturation. An increase of the endogenous Mos and Cyclin B1 protein was detected around the time an increased polysome loading was detected (Fig. 2D). At variance with previously published data^18^, only a marginal increase in Cyclin B2 protein accumulation could be detected in our experimental conditions.

Collectively the above findings indicate that the timing of translational activation in mouse oocytes follows GVBD in a manner distinct from that shown in *Xenopus* oocytes where translation of these mRNA is activated prior to GVBD^19^.

### The rates of degradation of Cyclin B1 and Cyclin B2 are similar

**Figure 3:**
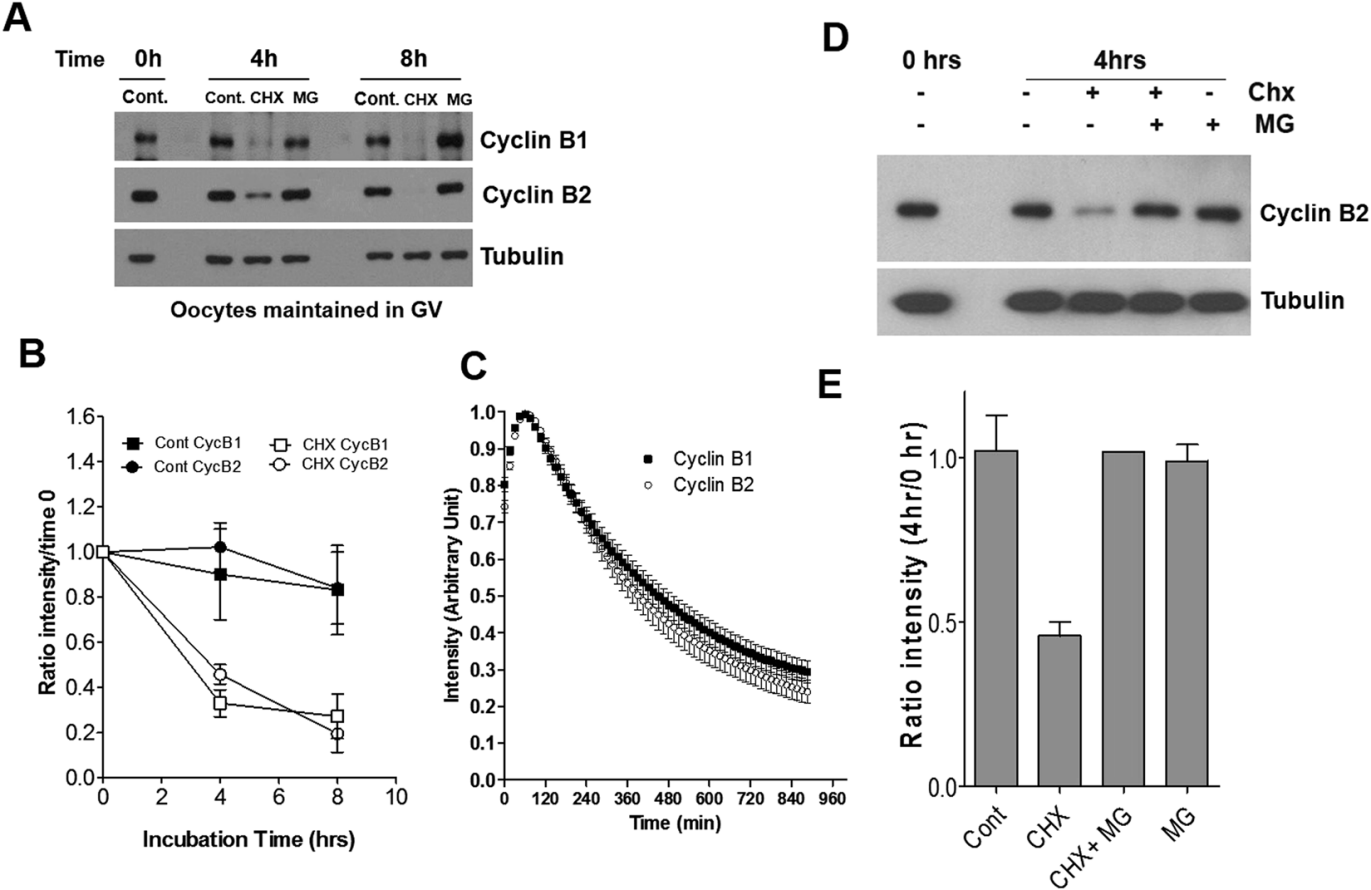
Cyclin B1 and B2 protein degradation rate in GV oocytes. (**A**) Oocytes (180 per lane) were cultured for 0, 4, or 8 h in milrinone-containing media (1 µM) with cycloheximide (20 µM) or MG132 (50 µM). The amount of Cyclin B1 and Cyclin B2 protein was monitored by Western blot with the antibodies described in the methods. Blotting for tubulin protein was used as the loading control. (**B**) The degradation rate was derived by measuring the density of the western blot bands after treatment with cycloheximide or MG132. Each point is the mean ± SEM, N = 3. (**C**) Oocytes were injected with constructs coding for fluorescent tagged Cyclins. After 2 h preincubation, cycloheximide was added and the fluorescence intensity of YFP tagged Cyclin B1 and B2 were monitored every 15 min. Data are the mean ± SEM of 21 oocytes for Cyclin B1 and 22 oocytes for Cyclin B2 from two independent experiments (**D**,**E**) Amounts of Cyclin B2 accumulated in GV oocytes treated with cycloheximide and MG132. The experiment was repeated three times with similar results.

Protein levels are dependent on the rate of both synthesis and degradation. Therefore, we examined whether differences in degradation rate would account for the different patterns of Cyclin B1 and B2 accumulation at the GV-to-prometaphase transition. It has been reported that Cyclin B1 and Cyclin B2 are degraded by proteasome after ubiquitination by ubiquitin ligase APC^cdh1^ in GV oocytes^18,23^. We then explored the difference in degradation rates of these proteins after treatment of cycloheximide, a protein synthesis inhibitor. As shown in Fig. 3A and B, Cyclin B1 and B2 levels decreased by approximately 50% after 4 h incubation with protein synthesis inhibitor, and became almost undetectable at 8 h. No major differences in rates of Cyclin B1 and Cyclin B2 degradation could be observed by this approach (Fig. 3B). Comparable rates of degradation of the two proteins was confirmed by injection of fluorescently tagged Cyclins (Fig. 3C). The possibility that these proteins decreased due to the toxicity of the cycloheximide treatment was ruled out because the level of tubulin protein was constant up to 8 h in the treated and untreated groups (Fig. 3A). A significant increase in Cyclin proteins could not be detected when oocytes were treated with the MG132 proteasome inhibitor. However, MG132 prevented the decrease in Cyclin B2 induced by cycloheximide (Fig. 3D,E). Together, these results suggest that the degradation rate of the two proteins is not a major factor in the divergent pattern of Cyclin accumulation in GV oocytes.

### The translation of Ccnb1 but not of Ccnb2 is dependent on CPEB

**Figure 4:**
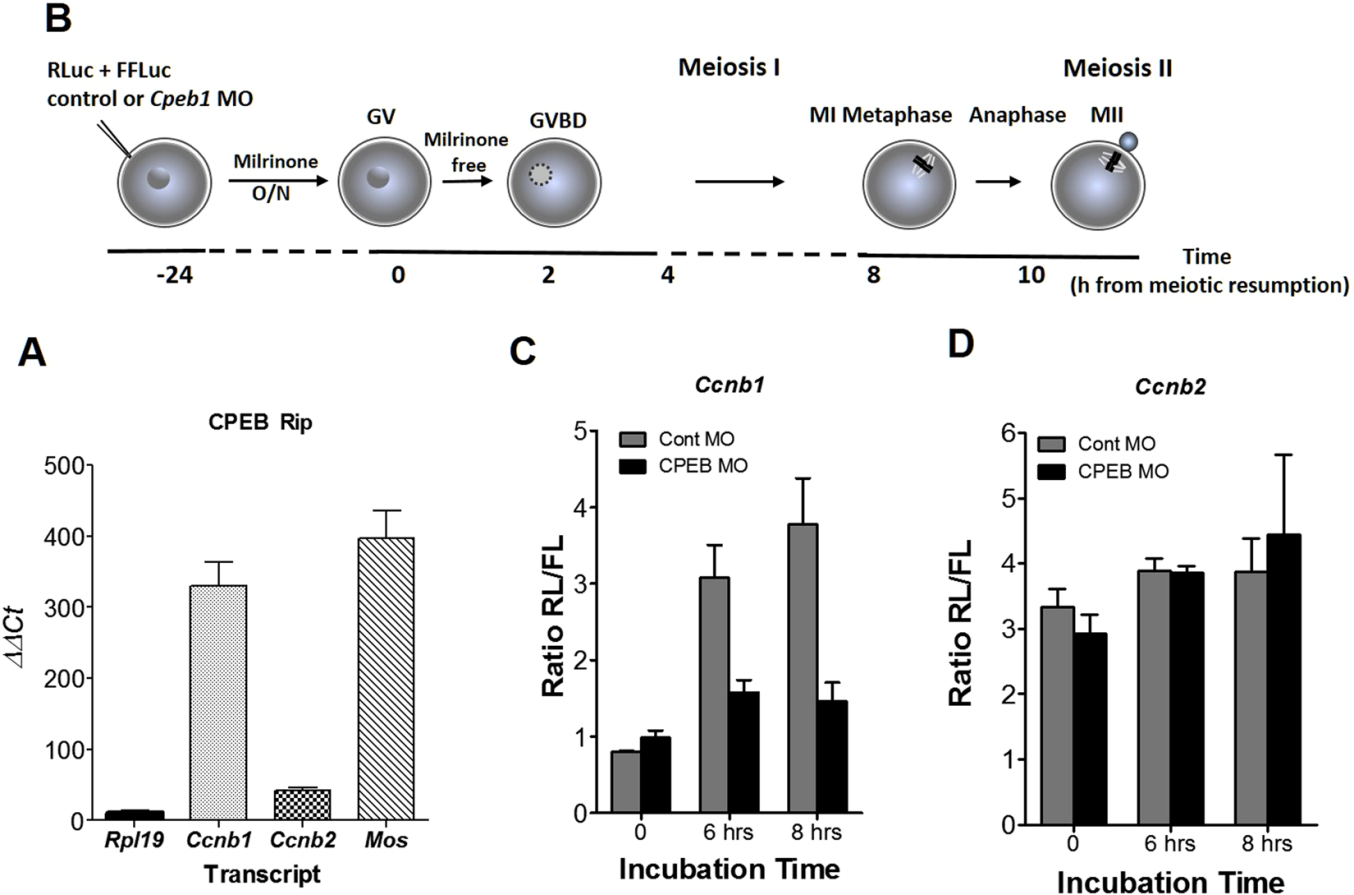
Cyclin reporter translation dependence on CPEB1. (**A**) CPEB binding to *Ccnb1*, *Ccnb2*, and *Mos* mRNAs. After immunoprecipitation of GV oocyte lysate with CPEB1 specific antibody, transcripts recovered in the pellet were reverse-transcribed followed by quantitative PCR with specific primers. The graphs report the mean ± SEM of three independent experiments. (**B**–**D**) CPEB1 was depleted from the oocytes by injection of morpholino oligomer (MO) or control MO. Either the *Ccnb1* or *Ccnb2* 3′UTR reporter was injected at the same time. After a 24 h incubation, milrinone was removed to induce cell cycle reentry, and the translation of *Ccnb1* (**C**) or *Ccnb2* reporter (**D**) was measured at 6 or 8 h after resumption of meiosis. The graphs are the mean ± SEM of three independent experiments.

Next, we explored the molecular basis for the differences in translational regulation between *Ccnb1* and *Ccnb2*. It is known that CPEB1 and its binding consensus site, cytoplasmic polyadenylation element (CPE), in the 3′UTR of mRNA is one of the determinants for the extent and timing of translation in *Xenopus* oocytes. Computational analysis of the 3′UTR of *Ccnb1* and *Mos* identified several putative CPEs, and it has been reported that both are canonical CPEB1 targets^19,24^. At least one CPE is present in 3′UTR of *Ccnb2* (Suppl. Figure 3). By RIP assay using a CPEB1 specific antibody followed by qPCR, we examined the association of these transcripts with CPEB. The transcripts of *Ccnb1* and *Mos* were significantly enriched in the immune complexes while the transcript of *Ccnb2* was borderline when compared to transcripts that do not possess CPEs (Fig. 4A). The *Rpl19* transcript that does not have putative CPE sequence and does not bind to CPEB was hardly immunoprecipitated by CPEB1 antibodies.

To verify the effect of CPEB1 on the translational regulation of *Ccnb1* and *Ccnb2*, CPEB1 protein was depleted by the injection of *Cpeb1* specific morpholino oligonucleotide (MO) (Fig. 4B) which effectively depletes CPBE1^20,22^. Upon depletion of CPEB1, the accumulation of *Ccnb2* reporter was not significantly affected 6 and 8 h after removal of milrinone (Fig. 4D). Conversely, the translation of *Ccnb1* reporter increased in oocytes injected with control MO as reported above (See Fig. 2B), but the accumulation of reporter in the *Cpeb1* specific MO injected group was significantly decreased (Fig. 4C). This latter effect is consistent with previous data monitoring the effect of CPEB1 depletion on ribosome loading onto the *Ccnb1* mRNA^22^. These results suggest that ribosome loading onto *Ccnb1* mRNA and translation of the transcript are modulated by CPEB1, whereas *Ccnb2* translation is largely independent of CPEB1.

### Changes in CPEB levels during oocyte maturation

**Figure 5:**
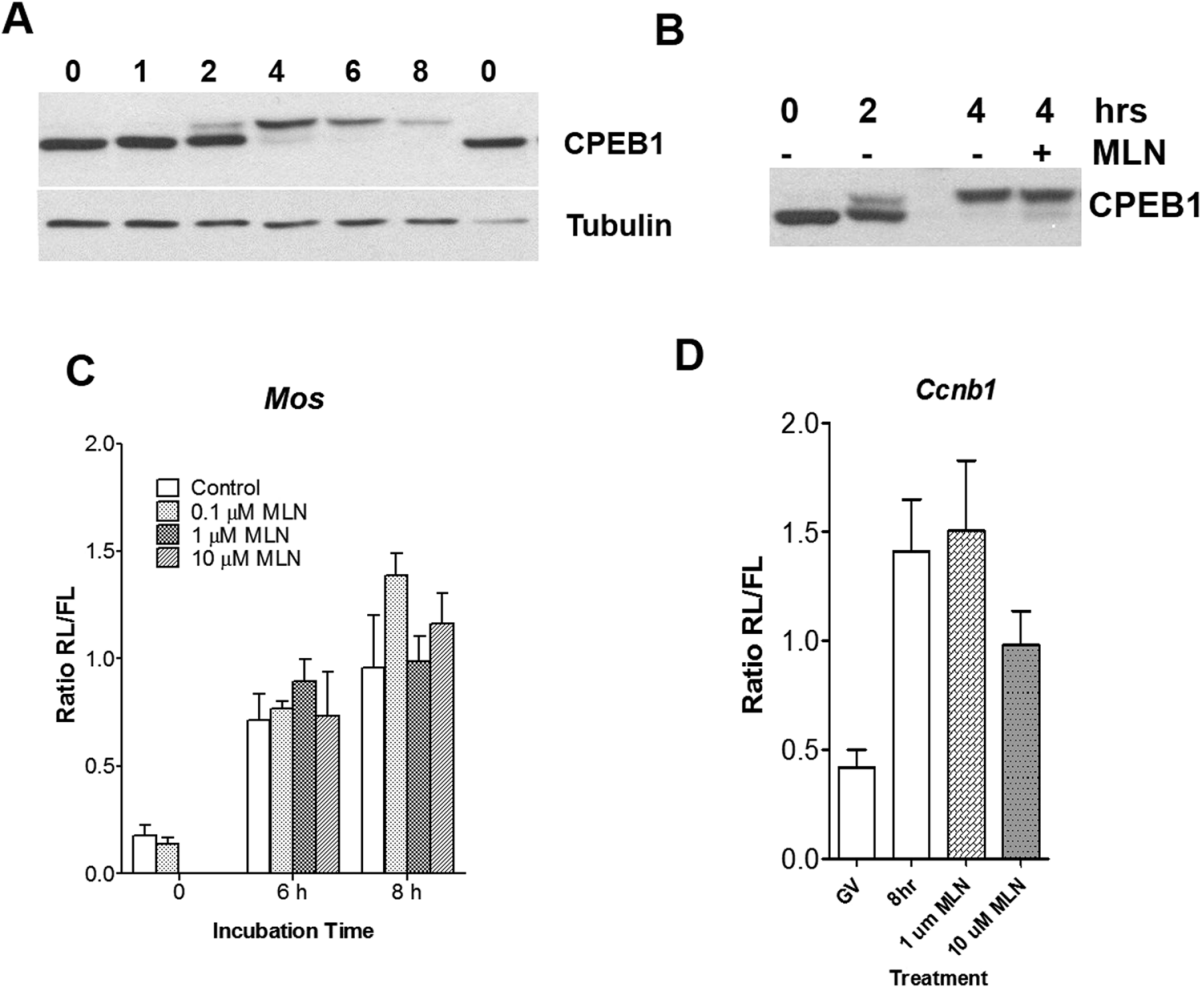
Effect of Aurora A kinase inhibitor on *Mos* and *Ccnb1* translation in mouse oocytes. (**A**) The phosphorylation state of CPEB1 during mouse oocyte maturation was monitored by western blot analysis. Extracts from 30 oocytes per lane were fractionated on the SDS PAGE gel, followed by transfer to the PVDF membrane and western blotting. A constant amount of protein application was confirmed by monitoring the amount of tubulin (lower panel). (**B**) CPEB1 phosphorylation during mouse oocyte *in vitro* maturation was not affected by Aurora A kinase inhibitor treatment. (**C**,**D**) Oocytes were injected with a *Mos* (**C**) or *Ccnb1* luciferase reporter (**D**) and matured in the absence or presence of the Aurora A kinase inhibitor, MNL8237. Oocytes were harvested at 6 or 8 h and the accumulation of luciferase was measured. To normalize the amount of injected cRNA, cRNA coding Firefly luciferase was used as a control and the data are expressed as a Renilla/Firefly luciferase ratio. The graphs are the mean ± SEM of three independent experiments. No significant differences between control and treated groups were observed.

It has been reported that CPEB is activated by phosphorylation by Eg2/Aurora A kinase during *Xenopus* oocyte maturation and this phosphorylation activates the translation of transcripts containing the CPE sequence^11^. However, some aspects of the CPEB role in mammalian oocyte maturation are less clear. To determine whether CPEB1 is phosphorylated during *in vitro* meiosis, we monitored the mobility shift of CPEB1 during mouse oocyte *in vitro* maturation in SDS-PAGE. The mobility of CPEB1 gradually decreased around 2 h after release from cell cycle arrest, and the amount of slow mobility form decreased afterward (Fig. 5A). This result is consistent with previous reports that CPEB1 is degraded after phosphorylation *in vivo* ^20,25–28^. More detailed western blots of CPEB shift in mobility during the first hour after milrinone removal revealed an additional early shift in mobility detected only when using Phos-tag gel (Suppl. Figure 4). This shift is concurrent with the initial activation of Cdk1 immediately before GVBD. However, this early event could not be related to changes in translation of the 3 transcripts (Fig. 2).

### Role of Cdk1 activation on translation of *Mos* and *Ccnb1*

The above data indicated that the major shift in mobility of CPEB1 occurs between 2 and 4 h after mouse oocyte reentry into meiosis. We then monitored whether the timing of this shift in mobility is compatible with changes in translation of *Mos* and *Ccnb1* reporters. At the same time we used Aurora A kinase inhibitor to determine whether this kinase was involved in activation of translation. Mouse oocytes were treated with an Aurora A kinase inhibitor (MLN8237) and the translation of a *Mos* luciferase reporter was monitored at 6 or 8 h after reentry into the cell cycle (Fig. 5C). This inhibitor effectively blocked Aurora A kinase activity because, in agreement with previous reports^29,30^, it completely prevents polar body extrusion (Suppl. Figure 5). However, inhibition of Aurora A kinase has no effect on the translation of *Mos* (Fig. 5C) or *Ccnb1* (Fig. 5D) reporters or on the shift in CPEB1 mobility (Fig. 5B).

**Figure 6:**
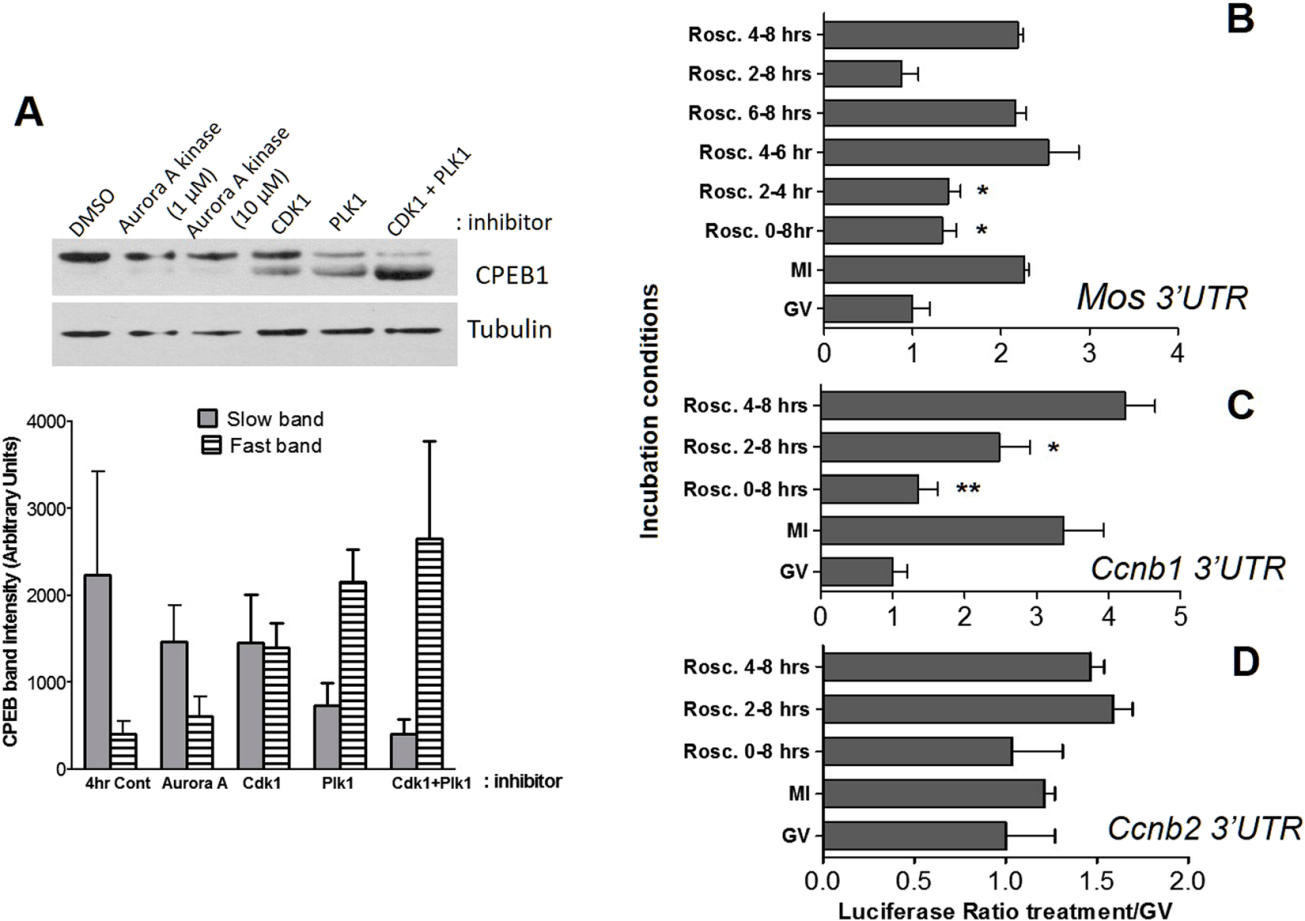
Cdk1 activity is necessary for translational activation after GVBD. (**A**) Effect of protein kinase inhibitors on the mobility shift of CPEB1. Loading was monitored by measuring the amount of tubulin (lower panel). The density of fast and slow migrating bands of CPEB1 from three independent blots were quantified and reported in the bar graph. N = 3. (**B**,**C**,**D**) Oocytes were injected with a *Mos* (**B**) *Ccnb1* (**C**) or *Ccnb2* (**D**) luciferase reporter and exposed to the Cdk inhibitor roscovitine (100 µM) for the entire 8 h incubation or for shorter time intervals reported in the ordinate. All oocytes were harvested at 8 h and the luciferase activity determined. Data are reported as the ratio of *Mos, Ccnb1 or Ccnb2* 3′UTR Renilla luciferase activity over Firefly luciferase activity used as a control for the injection. *Mos*: Bars are the mean ± SEM of 4 biological replicates. *CcnB1*: Bars are the mean ± SEM of 7 biological replicates. *CcnB2*: Bars are the mean ± range of 2 biological replicates *P < 0.05; **P < 0.01.

Further dissection of the effects of different inhibitors on the shift in mobility of CPEB1 showed that at 4 h the shift was partially blocked by treatment of Cdk1 inhibitor (roscovitine) or Plk1 inhibitor (BI2536) alone and almost completely prevented by a combination of two inhibitors (Fig. 6A). As expected, roscovitine added at the beginning of the incubation prevents GVBD completely (Rosc. 8 h) and consequently translation (Fig. 6B). However, when the inhibitor was added after 2 h, all oocytes reentered into GVBD. Note that only exposure to roscovitine between 2 and 4 h is sufficient to block translational activation of the *Mos* reporter, while the accumulation of reporter occurred normally when the inhibitor was added after 4 h (4–6 h, 6–8 h, and 4–8 h in Fig. 6B). Similarly, the translation of *Ccnb1* reporter was also affected only if roscovitine was added at 2 h even though the inhibition was not complete (Fig. 6C). Roscovitine had a marginal and non-significant effect on *Ccnb2* reporter translation (Fig. 6D). Taken together, these results strongly suggest that the major wave of translation, which in mouse oocytes occurs after GVBD in the window between 2 and 4 h, is directly or indirectly dependent on Cdk1 activation.

## Discussion

**Figure 7:**
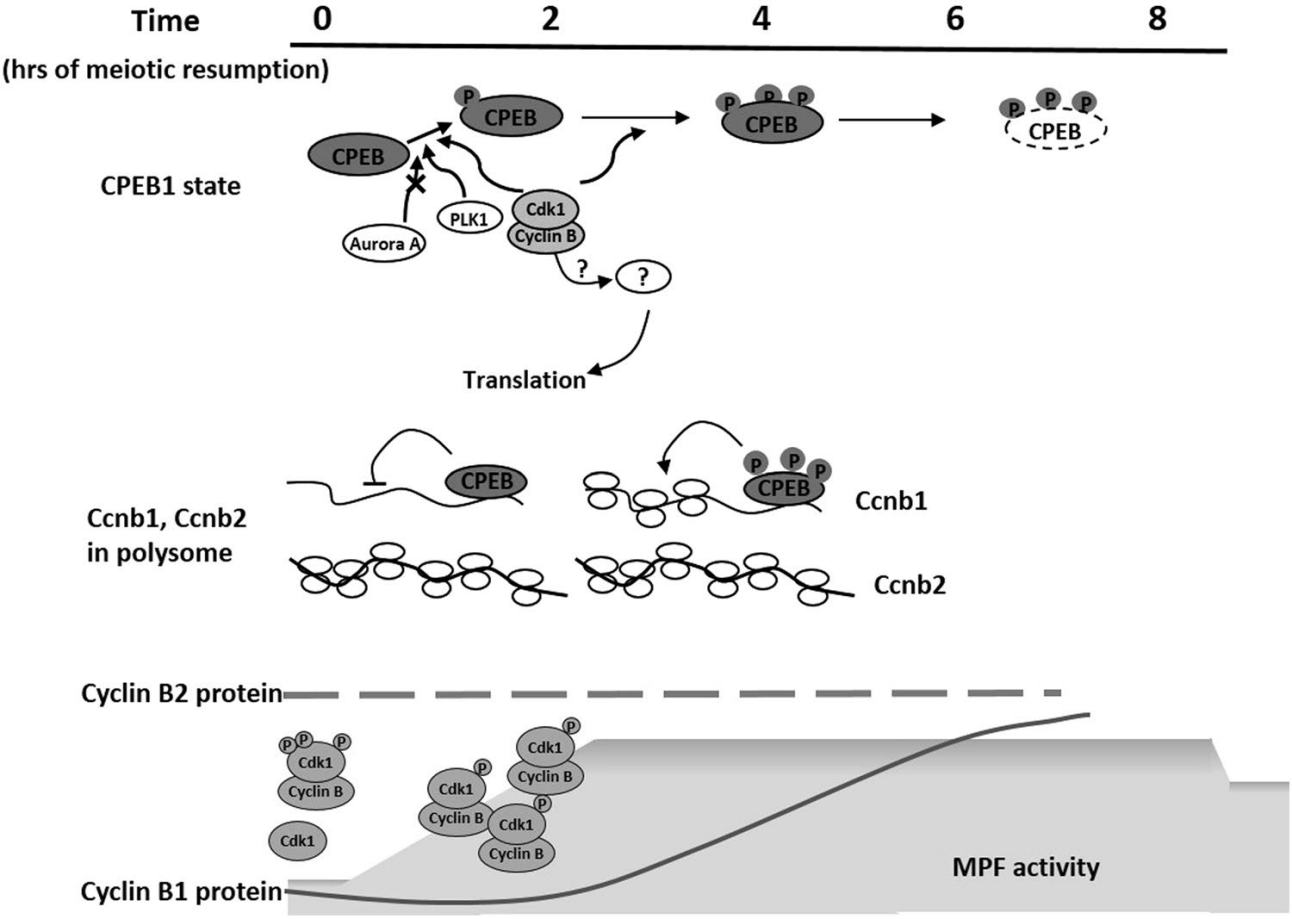
Proposed model for the regulation of translation of Cyclins during mouse oocyte maturation. Translation of endogenous Cyclin mRNAs was monitored by measuring ribosome loading onto the mRNAs or exogenously injected reporter accumulation. CPEB1 phosphorylation and reporter translation is independent of Aurora A kinase. It is instead directly or indirectly dependent on Cdk1 activation. Phosphorylated CPEB1 modulates the translation of *Ccnb1* whereas *Ccnb2* translation is largely independent of CPEB1. The rate of synthesis, rather than that of degradation, of the two proteins is a major determinant of Cyclin levels and of MPF levels in mouse oocytes.

Although the activation of an MPF complex in the female gamete of all species studied is essential for reentry into the meiotic cell cycle, different regulatory circuits have evolved to allow precise spatial and temporal MPF changes in tune with species-specific patterns of meitoic progression. In some species such as zebrafish (*Danio rerio*), Cdk1 but no Cyclin B proteins are intially present in GV oocytes^31^. These components will be synthesized only later during meiosis. In other species, several different Cyclin Bs are present in GV oocyte and are associated with Cdk1 to form an inactive pre-MPF complex. After hormone stimulation, *de novo* synthesis of several Cyclin Bs is required for GVBD in the *Xenopus* oocyte whereas mouse oocytes reeneter meiosis even in the presence of protein synthesis inhibitors^32,33^. These findings indicate differences in pre-MPF assembly and MPF regulation among different species. Regardless of these species differences, the translation of *Ccnb* mRNAs is consistently necessary for the modulation of Cdk1 activity and to support meiotic progression^34,35^. We show here that the steady state levels of the two major Cyclins present in mouse oocyte meiotic prophase is largely dependent on differential translation of the two corresponding mRNAs. Thus, the rate of translation contributes to maintaining a steady state of Cyclins essential for rapid reentry into meiosis. In addition, differences in the mode of translational activation determines the protein levels of the two Cyclins during meiotic progression (Fig. 7).

Given the finding that significant Cyclin degradation takes place in quiescent GV oocytes^18,36^, we posit that continuous synthesis is indispensable for maintaining Cyclin steady state. It has been proposed that Cyclin B2 degradation is fine-tuned through APC inhibition by Hec1^18^ while the Cyclin B1 rate of degradation is the balance of Cdh1-stimulated and Securin/Bub1b-inhibited APC activity^36^. Consistent with significant degradation rates in GV oocytes, APC loss of function by pharmacologic or genetic means induces a premature entry into meiosis^36–38^. Thus, the 50% decrease in Cyclins we detect at 4 h in our experiments (Fig. 3) implies that 50% of the Cyclin pool must be replenished by synthesis via mRNA translation. This finding indicates that significant rates of mRNA translation in GV oocytes are critical to maintain the stockpile of Cyclins necessary for rapid Cdk1 activation, and to establish a steady state permissive to reentry into meiosis. Of note, the degradation rates for Cyclin B1 we measured are virtually identical to those reported by others^16^. Within the time frame of our study, the proteasomal inhibitor MG132 treatment had a small or insignificant effect on the level of Cyclin B1 and Cyclin B2 proteins, unless protein synthesis is blocked. Some stabilizing effect was observed only at 8 h for Cyclin B1. Even though meiotic resumption induced by APC inactivation is slow and incomplete in our experimental model as well as in data reported by others^36^, all the above findings taken together indicate that a turnover of Cyclin is present in GV oocytes. The turnover inferred by the above measurements is likely slower than that measured in an extract of *Xenopus* MII oocytes^39^ where Cyclin has a very short half-life. A caveat in the interpretation of our data is that all of the measurements were done in the presence of protein synthesis inhibitors. Another possibility that needs to be taken into consideration is that a feedback mechanism linking degradation to translation maintains the appropriate steady states and is functioning in the oocyte. This implies that proteasome inhibition may also lead to a decrease in the rate of synthesis of Cyclins. The hypothetical factor(s) stabilized by proteasome inhibition and affecting translation is (are) at present unknown.

In frog oocytes, several Cyclins are differentially translated during the G2/M transition. Prior to GVBD, *Ccnb2* and *Ccnb*5 translation is activated first, followed by late translational activation of *Ccnb1* and *Ccnb4* during metaphase I. Among the three isotypes of Cyclin Bs (B1, B2, and B3) expressed in the female mouse germ cell^40^, two were detected by western blot in GV oocytes. We also show that *Ccnb3* mRNA is translated in GV oocytes but translation decreases dramatically with oocyte maturation (Fig. 1) and little signal could be detected by RiboTag IP. A recent report indicates that *Ccnb3* knockdown has no effect on GVBD but it may control anaphase onset in mouse oocytes^41^. Loss of function studies have provided conflicting results on the role of Cyclin B2 during mouse meiosis^42,43^, but a recent report using MO to deplete Cyclin B2 suggests its important role for the oocyte G2/M transition^18^. We found that *Ccnb2* mRNA is fully loaded with ribosomes in GV whereas *Ccnb1* is not (Fig. 1 and Suppl. Figure 1), and the 3′UTR reporter translation also shows a different pattern of translation rate in GV oocytes as well as after meiotic reentry (Fig. 2). In *Xenopus* oocytes, mRNA translation is activated before GVBD and is dependent on the function of CPEB. In our case, the translation of mouse *Ccnb2* occurs in the presence of low Cdk1, before GVBD and likely during oocyte growth (Fig. 2C), and in the absence of a phosphorylated CPEB (Fig. 5). Conversely, *Ccnb1* translation requires both. Thus, a different mode of regulation strongly suggests that while *Ccnb2* translation changes marginally, major increases in *Ccnb1* translation take place during prometaphase. Recent data from our laboratory provide further molecular explanation for the time-dependent rates of translation of *Ccnb1* during meiotic prophase by demonstrating the presence *Ccnb1* mRNAs with different 3′UTRs^44^.

In frog oocytes and for an initial burst of translation, Eg2 (Aurora A kinase) phosphorylates CPEB, followed by a second phase of Cdk1-dependent translation. The exact nature of the kinase involved in the initial activation in *Drosophila*, *Xenopus*, and bovine oocyte remains somewhat controversial^27,28,45–51^. Aurora A kinase inhibitors have no effect on CPEB phosphorylation, and depletion of Aurora A kinase does not inhibit early CPEB phosphorylation in frog oocytes^28^. Our pharmacological manipulations in mouse oocytes confirms that Aurora A kinase is likely not involved in CPEB1-mediated activation of translation up to MI (Figs 5,6,7); this finding is consistent with reports indicating a late Aurora A activation during mouse meiotic reentry^52^. Conversely, when Cdk1 activity is blocked with roscovitine, translational activation of the prototypic CPEB1 target mRNA, *Mos*, and *Ccnb1* is prevented (Fig. 6) and the shift in CPEB1 mobility is prevented. Of note, the time in which roscovitine is active in blocking reporter translation (2–4 h) is consistent with the timing of ribosome loading. Thus, in addition to clear differences in timing of translational activation between the mouse and frog, the involvement of different kinases needs to be reassessed in the oocyte of this species. In mouse oocyte, CPEB phosphorylation (inferred from the changes in electrophoretic mobility) requires Plk1 as well as Cdk1 because the pharmacological block of both kinases attenuated the CPEB1 shift in mobility in the SDS-PAGE (Fig. 6). We cannot exclude that the effect of the Plk inhibitor is indirect given the fact that this kinase is part of a positive feedback loop where it modulates Cdk1 activity by phosphorylation of CDC25 and Wee1, key regulators for Cdk1^53,54^. Although phosphorylation of CPEB1 by Cdk1 has been reported, indirect effects on CPEB1 phosphorylation state are also possible in the mouse. Consistent with previous reports^20^, a CPEB1 shift in mobility precedes destabilization of the protein (Fig. 5A). Mass spectrometry studies will be required to understand the exact phosphorylation pattern of CPEB1 in mouse oocytes during the different phases of the cell cycle.

In conclusion, the rate of translation of Cyclin B1 and B2 proteins, together with their degradation, is a major determinant of their divergent temporal accumulation during oocyte maturation. The Cdk1-independent translation of *Ccnb2* mRNA strongly suggest that this Cyclin is present in prophase and may play a critical role in setting the stockpile of Cyclin B protein for the assembly of pre-MPF. These findings are consistent with the recent report that Cyclin B2 plays a role during the G2/M transition in mouse oocytes^18^. Although translated at lower rate, Cyclin B1, which is traditionally considered the major contributor to the Cdk1 activity necessary for GVBD, is also present in GV oocytes. Later, during meiotic progression while Cyclin B2 levels change only marginally, the increasing Cyclin B1 protein between 2 and 4 h during prometaphase^42^ likely plays a  pivotal role to reinforce the Cdk1 activity and to drive cell cycle progression. Whether the two Cyclins impose distinct temporal and spatial controls on Cdk1 during meiotic entry and prometaphase remains to be determined.

## Methods

### Animal care and oocyte culture

The experimental procedures involving mice were approved by the University of California San Francisco Institutional Animal Care and Use Committee (Approval # AN163021-01C), and mouse care and use were performed in accordance with the relevant guidelines and regulations. Denuded oocytes were collected and microinjected in HEPES modified minimum essential medium Eagle (hMEM; Sigma-Aldrich, M2645) supplemented with 0.2 mM pyruvate, 75 μg/ml penicillin, 10 μg/ml streptomycin sulphate, 3 mg/ml BSA, 26 mM sodium bicarbonate and 2 μM milrinone (inhibitor of PDE3A to maintain the GV state). Oocytes were cultured at 37 °C under 5% CO_2_ in MEMα; minimum essential medium α (Gibco, 12561-056) supplemented with 0.2 mM pyruvate, 75 μg/ml penicillin, 10 μg/ml streptomycin sulphate and 3 mg/ml BSA with or without 2 μM milrinone.

### RiboTag immunoprecipitation

The C57BL/6-Zp3cre-RiboTag mice expressing the HA epitope tagged ribosomal protein L22 only in the oocyte were generated as previously described^22^. After isolation, oocytes were released from cell cycle arrest by removal of milrinone followed by collection at several time points up to metaphase. The immunoprecipitation of the HA-tagged ribosome complex was conducted as described in the previous reports^21,22^ followed by RNAseq or quantitative PCR.

### Morpholino antisense oligonucleotide microinjection

Isolated GV oocytes were microinjected with 5–10 pl of 1 mM morpholino oligonucleotides (GeneTools) against *Cpeb1* (5′-CTGCTTCTTCCAGAGAGAAAGCCAT-3′), or a standard control (5′-CCTCTTACCTCAGTTACAATTTATA-3′) using a FemtoJet express microinjector. After injection, oocytes were incubated overnight in MEMα containing 2 μM milrinone at 37 °C under 5% CO_2_.

### Reporter mRNA preparation and luciferase assay

The Renilla luciferase reporter (RL, Promega, E2241) was conjugated with the 3′UTR of mouse *Mos* (NM_0200212), *Ccnb1* (NM_172301) or *Ccnb2* (NM_007630.2) which are amplified with PCR with specific primer sets. Cloned RL and Firefly luciferase (FL) reporters were transcribed *in vitro* to generate cRNAs using the mMESSAGE mMACHINE T7 kit (Ambion, AM1344) and purified with RNeasy Plus Micro kit (Qiagen, 74034). The injection control Firefly luciferase cRNA was polyadenylated with the Poly(A) tailing kit (Ambion, AM1350). After 5–10 pl of cRNAs (12.5 ng/μl) injection into the oocyte at a 1:1 ratio, luminescent reaction was assessed with the Dual Luciferase Reporter Assay kit (Promega, E1910) and detected with the SpectraMaxL Luminometer (Molecular Devices). Data are reported as the ratios of the luminescence of the Renilla luciferase reporter to that of Firefly luciferase. For the effect of various inhibitors on translation, milrinone was washed out and the oocytes were incubated with various inhibitors for the indicated times.

### RNA Immunoprecipitation (RIP) and Real-time qPCR

For the RNA IP (RIP) with CPEB1, collected oocytes in RNase-free PBS with 0.1% polyvinylpyrrolidone were incubated in RIP lysis buffer (30 mM Tris-HCl pH 7.4, 150 mM NaCl, 10 mM MgCl_2_, 0.5% NP-40, 0.25 mM Na_3_VO_4_, 10 mM β-glycerolphosphate, 1 mM dithiothreitol, protease inhibitors, 40 U RNAseOUT, vanadyl ribonucleotide and 100 μg/ml cycloheximide) and spun down. CPEB1 antibody or mouse IgG (Abcam, ab37355) was added to the supernatant of lysed oocyte and incubated for overnight at 4 °C on a rotor. 25 μl of pre-washed protein G sepharose (Invitrogen, 101243) were added and incubated for 5 h at 4 °C on a rotor. Beads were washed with wash buffer (30 mM Tris-HCl pH 7.4, 200 mM NaCl, 10 mM MgCl_2_, 0.5% NP-40, 0.25 mM Na_3_VO_4_, 10 mM β-glycerolphosphate, 1 mM dithiothreitol, protease inhibitors, 40 U RNAseOUT and 1 M Urea) five times. RNA extraction was performed using the RNeasy Plus Micro Plus kit and extracted RNA was used to prepare cDNA using the SuperScript III First-Strand Synthesis system (Invitrogen, 18080-051) with random hexamer oligonucleotide primers. Real-time qPCR was performed using KAPA SYBR FAST ABI Prism 2 × qPCR master mix (Kapa Biosystems, KK4603) in an ABI 7900 Real-Time PCR system (Applied Biosystems). Fold-enrichment was calculated using the 2^−ΔΔCt^ method (Livak and Schmittgen, 2001).

### Western blot

Collected oocytes at indicated times of incubation under maturing conditions were extracted in SDS-PAGE loading buffer with a cocktail of phosphatase and protease inhibitors (PhosSTOP, Roche). The lysates were resolved on 8% polyacrylamide gels and transferred to a PVDF membrane. CPEB1 (Abcam, ab73287), Mos (LS-C164489, LifeSpan BioSciences, Inc), Cyclin B1 (4135, Cell Signaling Tech.) or Cyclin B2 (AF6004, R&D Systems) antibody was used for immunoblotting. An antibody against α-tubulin (T6074, Sigma-Aldrich) was used as loading control.

### Monitoring of protein degradation

YPet coding sequence was cloned downstream of *Ccnb1* or *Ccnb2* coding sequence. To generate cRNAs, *Ccnb1* and *Ccnb2* reporters were transcribed *in vitro*, polyadenylated and purified. Oocytes were injected with 5–10 pl (150 ng/μl) of either *Ccnb1* or *Ccnb2* reporters and incubated for 2 hours in the presence of 1 μM of Dinaciclib (Selleckchem, SCH727965), a specific Cdk1 inhibitor, to maintain the oocytes arrested at GV stage. Oocytes were then incubated with 20 μg/ml of cycloheximide and the amount of fluorescent Cyclin B1 or B2 was recorded in time lapse microscopy every 15 min using Nikon Eclipse inverted microscope. The fluorescence signals were normalized to the signal at the beginning of the recording.

All data were analyzed and plotted using the Graph PAD Prism software package.

## Electronic supplementary material

Supplementary Information
