# Supplementary material for: The Translation of Cyclin B1 and B2 is Differentially Regulated during Mouse Oocyte Reentry into the Meiotic Cell Cycle
Source: Sci Rep. 2017 Oct 26;7:14077. doi: 10.1038/s41598-017-13688-3 (PMC5658433; doi:10.1038/s41598-017-13688-3)
Supplement: Supplementary file 1 — Supplementary Information [file 41598_2017_13688_MOESM1_ESM.pdf]

Supplemental figures **Han *et al.***

The Translation of Cyclin B1 and B2 is Differentially Regulated during Mouse Oocyte Reentry into the Meiotic Cell Cycle

Seung Jin Han[1,*], João Pedro Sousa Martins[2], Ye Yang[2,3], Min Kook Kang[1], Enrico Maria Daldello[2], Marco Conti[2,*]

[1] Department of Biological Sciences, Inje University, Gimhae, 50834, Republic of Korea

[2] Center for Reproductive Sciences, University of California, San Francisco, CA 94143, USA Eli and Edythe Broad Center of Regeneration Medicine and Stem Cell Research, University of California, San Francisco, CA 94143, USA Department of Obstetrics and Gynecology and Reproductive Sciences, University of California, San Francisco, CA 94143, USA.

[3]State Key Laboratory of Agrobiotechnology, College of Biological Sciences, China Agricultural University, Beijing 100193 People's Republic of China

*Corresponding author e-mail: hansjin@gmail.com

*Co-corresponding author e-mail: contim@obgyn.ucsf.edu

Suppl. Fig.1

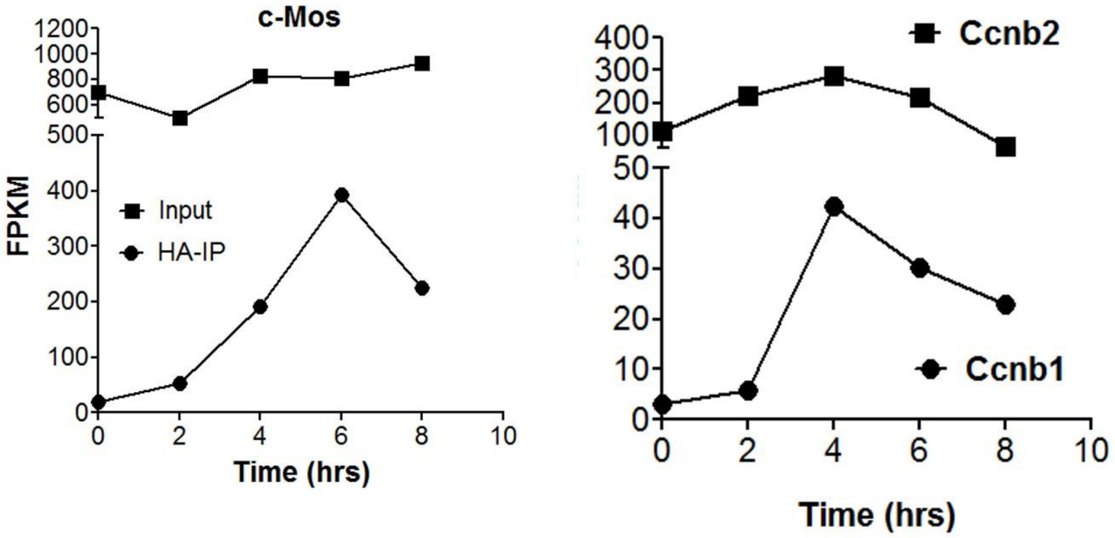

Figure 1. Reads for *Mos, Ccnb1* and *Ccnb2* in the RiboTag RIP. Oocytes from RiboTag transgenic mice were collected at the indicated time points after release from cell cycle arrest and immunoprecipitation was performed with HA antibody to collect transcript-associated ribosomes. After purification and amplification of the co-precipitated mRNA, the pool of amplicons was sequenced. The corrected number of reads in the IP pellet (FPKM, fragments per kilobase of exon per million reads) for *c-Mos* (left graph), *Ccnb1* and *Ccnb2* (right graph) sequence are reported.

Suppl. Fig.2

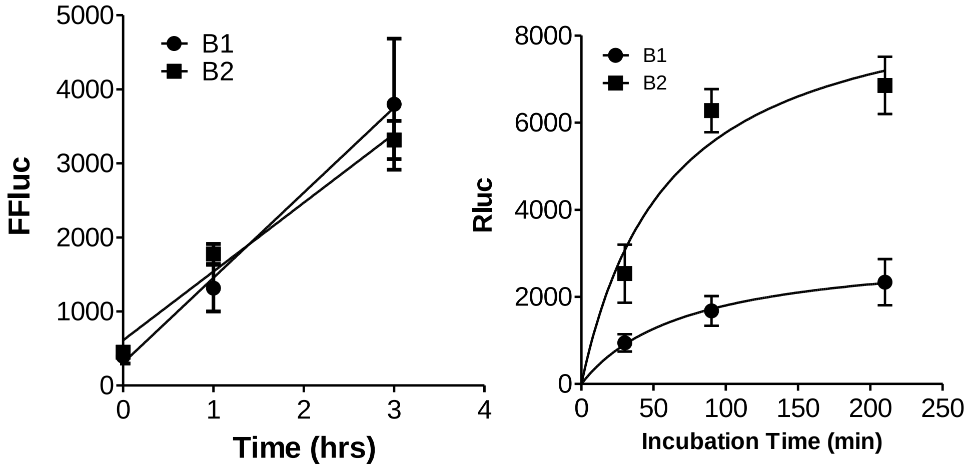

Figure 2. Rate of translation of the *Ccnb1* and *Ccnb2* reporters. *Ccnb1* or *Ccnb2* reporter cRNA were injected in GV oocytes and the rate of reporter accumulation was measured from 30 min after injection at the indicated time points. The left graph shows the translation of Firefly luciferase as a control. The Renilla luciferase levels were not corrected for the co-injected Firefly luciferase

Suppl. Fig.3

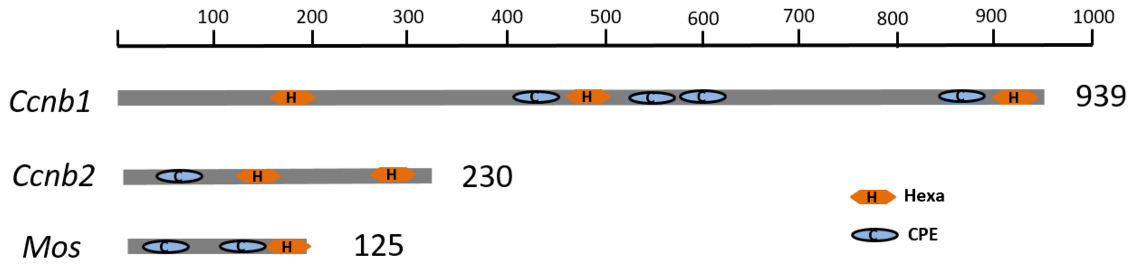

Figure 3. 3' untranslated region (3'UTR) motif analysis of the *Ccnb1*, *Ccnb2* and *Mos* gene for mouse. The putative cytoplasminc polyadenylation element (CPE) and hexanucleotide polyadenylation signal (Hexa) are indicated.

Suppl. Fig.4

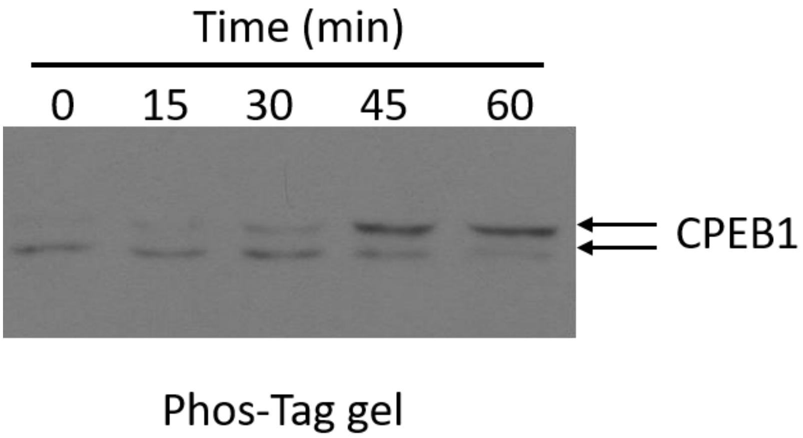

Figure 4. CPEB1 phosphorylation during early mouse oocyte maturation. The phosphorylation of CPEB1 was monitored using a phos-tag gel system. This system enhances the mobility shift degree of phosphorylated protein in the SDS-PAGE (Kinoshita et al., 2006). The early mobility shift of phosphorylation of CPEB1 was monitored during 1 h after the resumption of meiosis.

Suppl. Fig.5

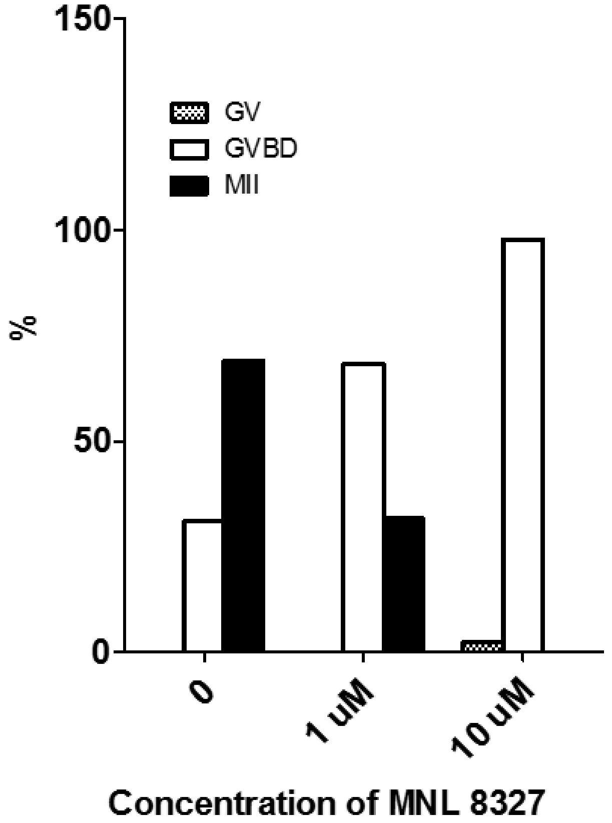

Figure 5. Inhibition of Aurora A kinase activity prevents polar body extrusion. The GV oocytes were released from cell cycle arrest by removal of milrinone and incubated with or without Aurora A kinase inhibitor, MNL8237. The GV, GVBD and polar body extrusion oocytes were counted at 16 h.

Suppl. Fig.6

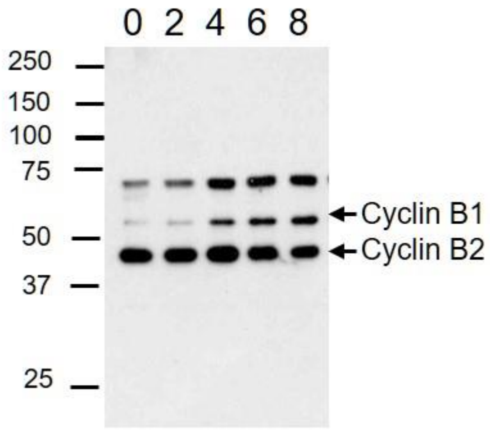

Fig. 2D. Cyclin B1. The blot was re-probed with Cyclin B1 specific antibody without stripping after incubation with Cyclin B2 antibody

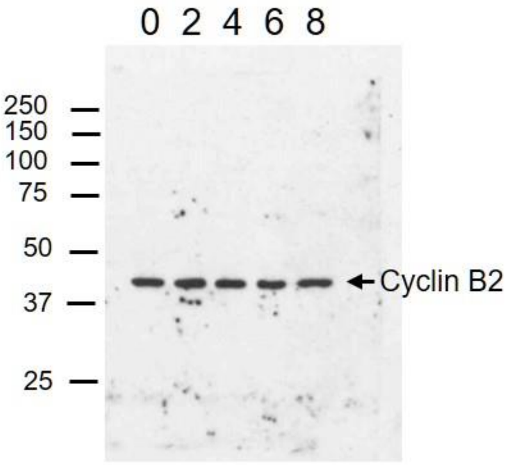

Fig. 2D. Cyclin B2

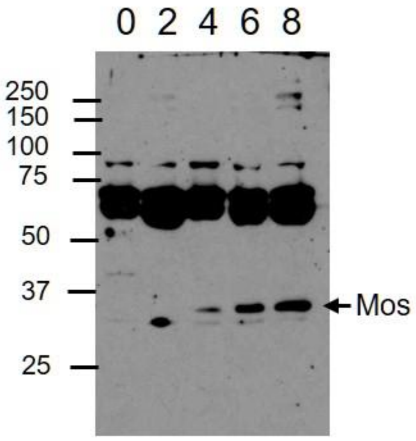

Fig. 2D. Mos

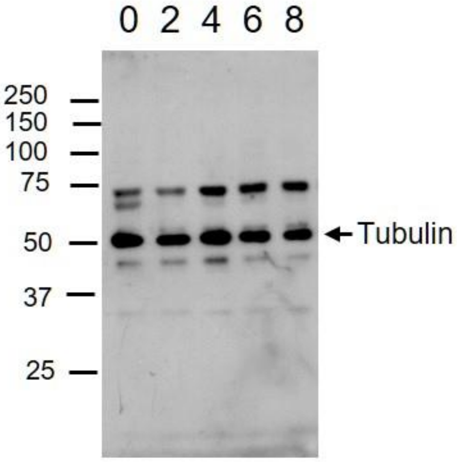

Fig. 2D. Tubulin

Suppl. Fig.6

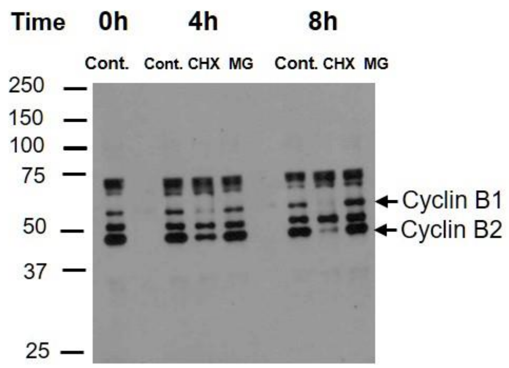

Fig. 3A. Cyclin B1. The blot was re-probed with Cyclin B1 specific antibody without stripping after incubation with Cyclin B2 antibody

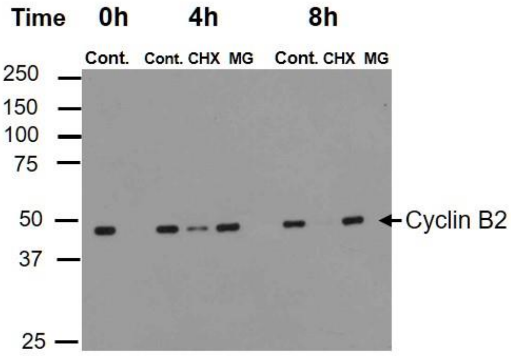

Fig. 3A. Cyclin B2

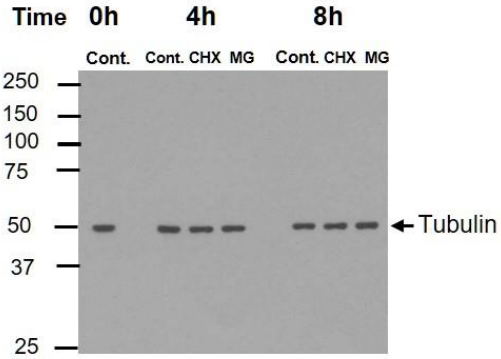

Fig. 3A. Tubulin

Suppl. Fig.6

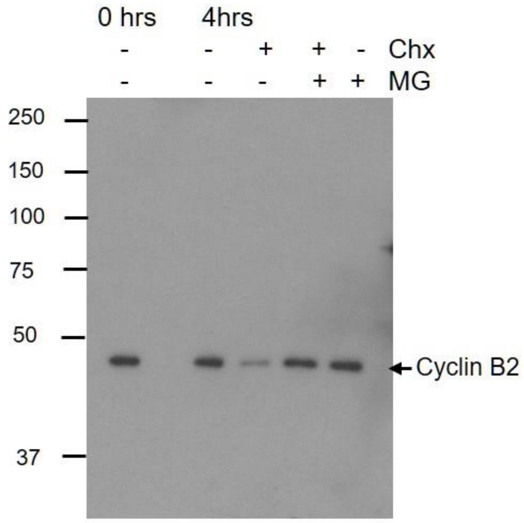

Fig. 3C. Cyclin B2

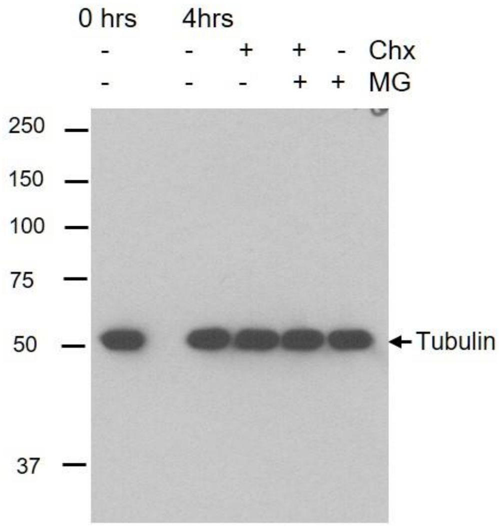

Fig. 3C, Tubulin

Suppl. Fig.6

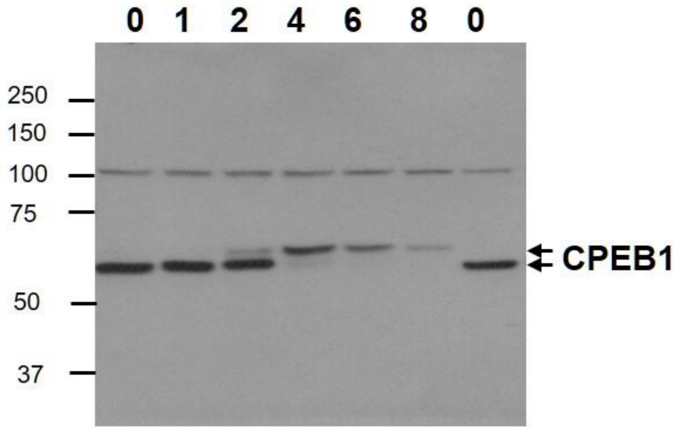

Fig. 5A. CPEB

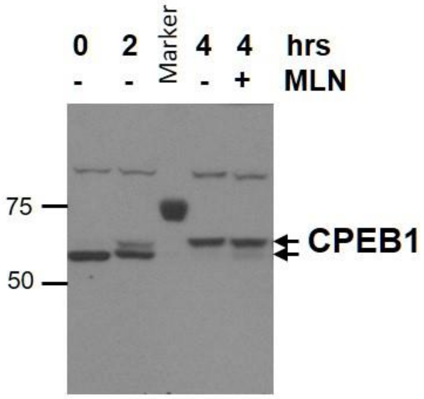

Fig. 5B. CPEB

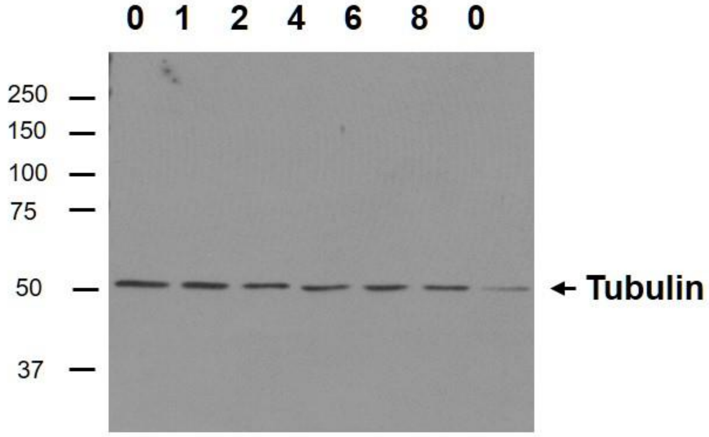

Fig. 5A. Tubulin

Suppl. Fig.6

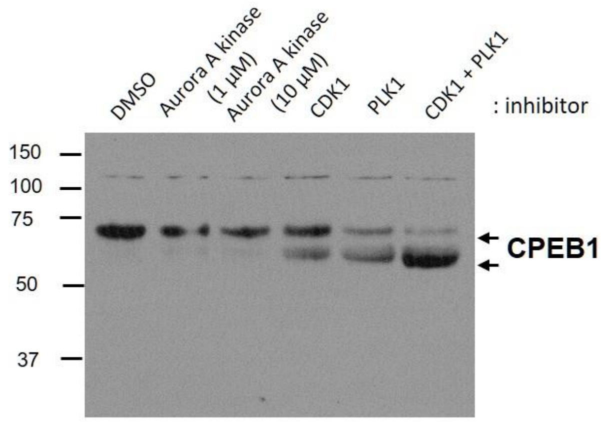

Fig. 6A. CPEB

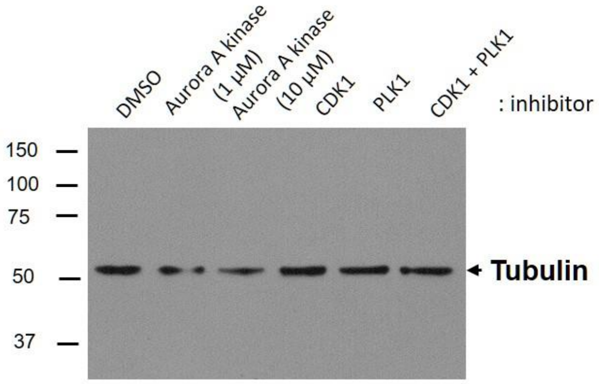

Fig. 6A. Tubulin

Figure 6. Uncropped images of panels in main figures.
